# Vulnerable Narcissism in Social Networking Sites: The Role of Upward and Downward Social Comparisons

**DOI:** 10.3389/fpsyg.2021.711909

**Published:** 2021-09-14

**Authors:** Fanchang Kong, Meiru Wang, Xingjie Zhang, Xiaoyao Li, Xiaojun Sun

**Affiliations:** Key Laboratory of Adolescent Cyberpsychology and Behavior, Ministry of Education, School of Psychology, Central China Normal University, Wuhan, China

**Keywords:** social networking sites, vulnerable narcissism, social comparison, parallel mediate, social media

## Abstract

Social networking sites (SNSs) have provided a new platform for people to present their narcissism. The objective of the current study was to investigate the underlying mechanisms between active and passive SNS use and vulnerable narcissism among college students. In achieving this, the study based its method on the media effect and social comparative theory and recruited 529 participants to complete the Surveillance Use Scale, Iowa–Netherlands Comparison Orientation Measure, and Hypersensitivity Narcissistic Scale. The results showed that active and passive SNS use were positively related to upward and downward social comparisons. Active and passive SNS use also indirectly predicted vulnerable narcissism through the parallel mediation of upward and downward social comparisons. This study also revealed the vital role of social comparison in the association between SNS use and vulnerable narcissism.

## Introduction

Narcissism, as a dimensional personality trait, reflects an inflated self-concept and behaviors that intend to maintain this self-concept in the face of reality (Morf and Rhodewalt, 2001). Early research has distinguished two forms of narcissism, namely, the grandiose and vulnerable subtypes (Wink, 1991). These subtypes are significantly different despite their shared commonalities, which refer to feelings of superiority and antagonism, in essence (Krizan and Herlache, 2018). In particular, grandiose narcissism is characterized by overt confidence, extraversion, and dominance, whereas vulnerable narcissism reflects high emotional sensitivity, defensiveness, and the expectation of attention (Miller et al., 2011). In comparison with grandiose narcissism, vulnerable narcissists tend to report higher interpersonal distress (Dickinson and Pincus, 2003), have poorer cognitive flexibility (Ng et al., 2014), and rely more on social feedback mechanisms to regulate themselves (Zeigler-Hill et al., 2008). In social networks, people with grandiose narcissism often make positive self-disclosures in communication, while those with vulnerable narcissism use more objective and indirect communication methods to fulfill their needs (Ozimek et al., 2017). Thus, the emergence of social networks has provided a platform for vulnerable narcissists to express themselves. For instance, studies have found a close relationship between Facebook use and vulnerable narcissism (Ozimek et al., 2018).

A recent study found that over 989 billion Chinese people exhausted ample time communicating on social networking sites (SNSs) by the end of 2020, with most of these users being teenagers (CNNIC, 2021). The internet became a social environment that could boost positive self-views and had the prime ability to integrate into the lives of people (Twenge, 2013). SNSs, in particular, provide users with unique platforms that allow them to share their information through personalized web pages and interact with others using the internet. These characteristics enable many people to use SNSs to satisfy their need for self-expression (Nadkarni and Hofmann, 2012). However, research has found that narcissists may use the internet to gain admiration and recognition (Dickinson and Pincus, 2003). On the one hand, “weak tie” network platforms, i.e., less reciprocal platforms that lack close emotional support such as Twitter, satisfy the need of vulnerable narcissists to get the attention of many users while averting direct communication (Bergman et al., 2011). On the other hand, the asynchrony of the internet allows individuals to elaborate their information, subsequently increasing their psychological need to get feedback (Hendin and Cheek, 1997). Nonetheless, the characteristics of social networks may also affect individual preferences according to media effects (Valkenburg et al., 2016). Therefore, in addition to the characteristics of the Internet use of narcissists that have been emphasized in previous studies, e.g., Ng et al. (2014), we should also look at the effects of SNSs on individuals in the context of such a considerable number of users. Thus, this study intended to explore the relationship between SNSs and individual preferences vis-à-vis vulnerable narcissism.

### SNSs Use and Vulnerable Narcissism

The relationship between SNS use and narcissism has been explored in previous studies, most of which indicate that high levels of narcissism have the tendency to lead to intensive SNS use (Bergman et al., 2011; Gnambs and Appel, 2018). Despite this, the continuous SNS usage of narcissists remains a question. According to the model of “reinforcing spirals” (Slater, 2007), SNS use and narcissism may interact with each other, which leads to the formation of a cross-lagged process. For example, the 1-year longitudinal study by Halpern et al. (2016) found that the frequency of taking selfies of narcissist individuals is directly correlated with the increase in selfie production, which can also raise levels of narcissism over time. Another study by Trepte and Reinecke (2013) confirmed the interaction between SNSs and individual self-disclosure traits, wherein individuals with high self-disclosure traits are more inclined to use SNSs and have more frequent social interactions. Website usage activities also increase the tendency of individuals to self-disclose online. In contrast, media effects or socialization effects reveal that spending time on SNS profiles causes young people to endorse more positive self-views (Gentile et al., 2012). In discussing the relationship between SNSs and vulnerable narcissism, there are at least two directions worthy of our consideration. On the one hand, SNSs provide vulnerable narcissists with opportunities for positive self-presentation. Unlike grandiose narcissists, vulnerable narcissists exhibit low extroversion, which means they are more likely to avoid social activities and appear to be more introverted or withdrawn from the attention of others (Pincus and Lukowitsky, 2010). Thus, SNSs are their “outlets” and tools for self-presentation, as these sites allow individuals to present self-enhancing content on their homepages, wall posts, and status updates (Kauten et al., 2015). In this way, individuals are encouraged to gradually internalize their perfect self-images that were carefully constructed on the internet, which, in turn, promotes a more positive self-concept (Walters and Horton, 2015). On the other hand, vulnerable narcissists have access to supportive resources coming from SNSs. Vulnerable narcissists are usually highly sensitive to the opinions of others, especially when it comes to negative evaluation. Conversely, they are eager to seek positive reviews such as recognition and praise (Pincus and Lukowitsky, 2010). SNSs encourage users to respond positively to the information of other people, which is why most of the comment sections in SNSs are active (Greitemeyer et al., 2014). This continuous positive feedback can enhance the superiority of an individual and further enhance their self-concept (Gentile et al., 2012), which is the cognitive cornerstone of the narcissistic system (Walters and Horton, 2015).

The utilization of SNSs can be dichotomized into active and passive SNS use (Burke et al., 2010; Verduyn et al., 2015). SNSs provide opportunities for vulnerable narcissistic individuals to promote themselves. Vulnerable narcissists tend to be afraid of their relationships with others because they are protecting themselves from shame and potential negative evaluation during their search for admiration (Casale and Fioravanti, 2018). Conversely, they are more likely to have a stronger preference for online social interactions using platforms with less reciprocation (Casale et al., 2016). These behaviors displayed by vulnerable narcissists may be passively perceived by others, with these passive behaviors in social networks being called passive SNSs. The passive use of SNSs indicates that the communication behavior of an individual only involves browsing information and without necessarily having direct exchanges with other individuals, e.g., viewing the dynamics of others and browsing their web page recommendations. On the other hand, the active use of SNS includes activities that promote communication, e.g., posting status updates and commenting on the moments of others (Burke et al., 2010). Furthermore, various forms of SNS use may affect users differently. For example, active SNS use is often associated with increased life satisfaction (Kim and Lee, 2011) and decreased negative feelings (Fardouly et al., 2015). However, the passive use of SNS negatively predicts depression (Tandoc et al., 2015) and self-esteem (Liu et al., 2017). As pointed out in a study by Panek et al. (2013), future studies about SNS use and personality traits must distinguish between different types of SNSs and different types of use. Therefore, exploring the influence of SNS use on individuals by combining active and passive use is essential.

### Mediating Role of Social Comparison

According to previous studies, if an indirect effect does not receive proper attention, the relationship between two variables of concern may not be fully considered (Raykov and Marcoulides, 2012). Social comparison is a universal phenomenon in human social life, but the convenience and immediacy of social networks make these social comparisons happen instantly. This means that social networks have become an important place for individual social comparisons (Coyne et al., 2017) in different ways. First, individuals can obtain information from others, make social comparisons, and influence their self-evaluation process through social networks anytime and anywhere (Vogel et al., 2014). Second, social comparison is an important means of individual self-enhancement. Specifically, individuals obtain self-evaluation information by comparing what they have with what others possess (Festinger, 1954). Therefore, it is important to understand the functions of social comparison during the use of SNSs.

The social comparison indicates that people define their social traits through comparisons with others (Xing and Yu, 2005). Social comparison can be divided into three types according to its directions. The first is the parallel direction, where the comparison takes place between people with similar levels. The second is the downward direction, which means the comparison happens between the individual and the people who are inferior to them. The third is the upward direction, where the comparison occurs between the individual and the people who are superior to them. Some characteristics of SNSs, e.g., asynchrony and multiple audiences, make it an ideal platform for social comparisons (Lee, 2014). Specifically, active SNS use can stimulate individuals to make upward social comparisons. Given that we always hope to be better than others when the attitudes of people expressed on SNSs collide with the ideas of others, individuals can realize their shortcomings and continuously improve themselves after marking upward comparisons. In addition, people may prefer to browse information to express their opinions on SNSs (Rozgonjuk et al., 2019). Some researchers believe that passive SNS use can predict upward social comparisons (Burnell et al., 2019; Hu and Liu, 2020), which can be induced in individuals by viewing good information (Chou and Edge, 2012). A study by McEwan (2013) claimed that the passive use of social media is done to reduce uncertainty and seek approval. Moreover, another study by Alicke and Govorun (2005) proposed the better-than-average effect, in which people believe that they perform better in many aspects compared with most people.

Contrary to the discussed ideas on the upward comparison, once a user experiences negative emotions after a social comparison, the desire to maintain a positive self could encourage them to adjust the level of a downward comparison (Gong and Zhang, 2020). Thus, people end up spending more time editing and revising the information to be presented to gain the approval of others. Additionally, the “like” and “comment” features on SNSs may encourage individuals to make downward social comparisons after enhancing their senses of superiority and privilege. Downward social comparisons on SNSs can significantly predict individual vulnerable narcissism (Kong et al., 2020). Furthermore, social comparison theory states that self-evaluation is gradually formed in the process of comparing with others (Festinger, 1954). The downward social comparison also improves individual satisfaction, self-esteem, and self-evaluation, which are important characteristics of narcissists (Foddy and Kashima, 2002). Conversely, users might think that other people have better and happier lives because of their good individual images created on SNSs in the process of upward social comparison (Chou and Edge, 2012). In turn, this might reduce their self-evaluation level (Appel et al., 2015).

Therefore, upward and downward social comparisons have probable effects on levels of self-evaluation. In particular, as a subordinate concept of narcissism, vulnerable narcissism may be influenced by social comparisons. Ultimately, the objective of the current study was to provide a deeper understanding of the relationship between the two forms of SNS use and vulnerable narcissism. It also aimed to explore the role of social comparison among these two variables. Based on the related literature discussed in the previous sections, the present study postulated the following hypotheses:

- Hypothesis 1a: Active SNS use positively predicts vulnerable narcissism.
- Hypothesis 1b: Passive SNS use positively predicts vulnerable narcissism.
- Hypothesis 2a: Upward and downward social comparisons mediate the relationship between active SNS use and vulnerable narcissism.
- Hypothesis 2b: Upward and downward social comparisons mediate the relationship between passive SNS use and vulnerable narcissism.

**Figure 1:**
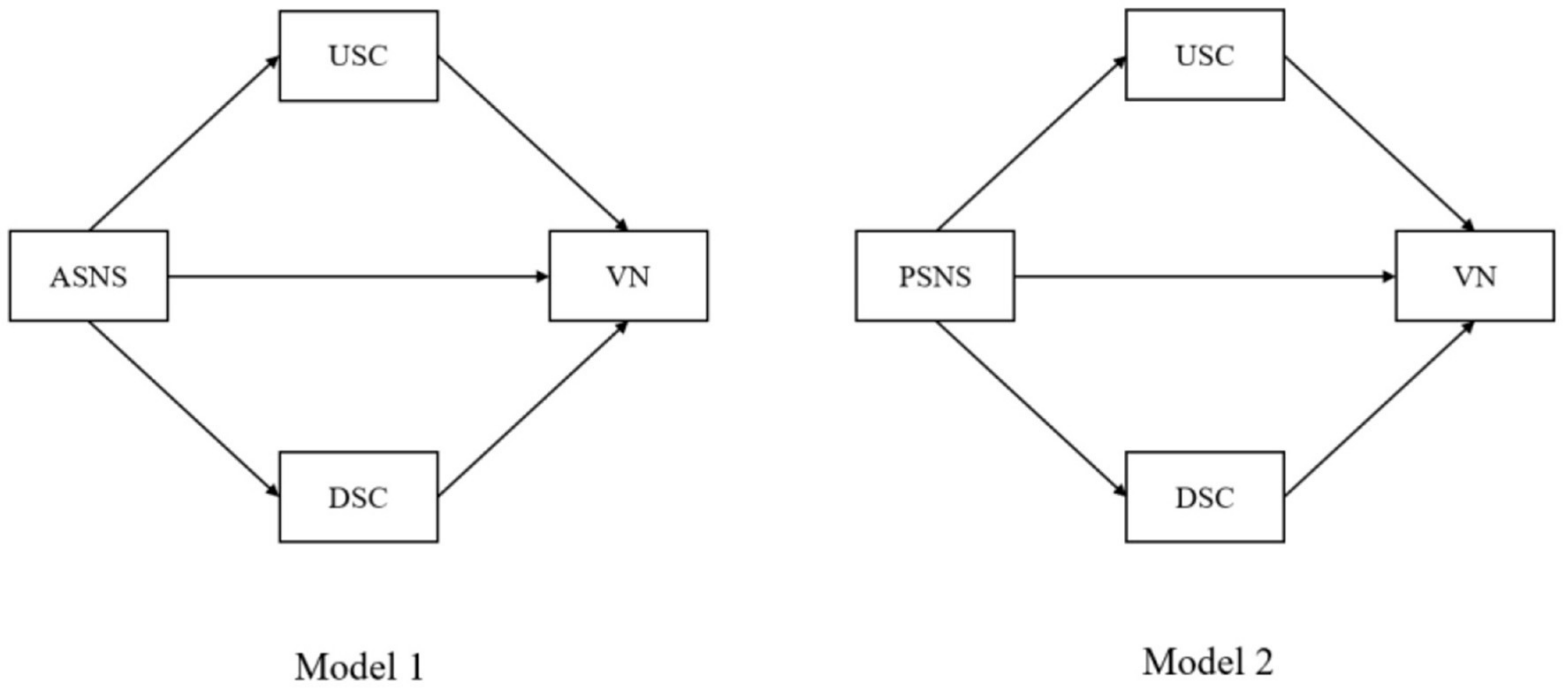
Conceptual model. ASNS, Active social networking sites; PSNS, Passive social networking sites; USC, Upward social comparison; DSC, Downward social comparison; VN, Vulnerable narcissism.

The overall conceptual model is displayed in Figure 1.

## Method

### Participants

The study used the pwrSEM app before conducting the investigation to make an *a priori* power analysis for the mediation analysis according to the contributions of a study conducted by Wang and Rhemtulla (2021). We set the regression coefficient of each path to 0.3, which represents the moderate effect size. When the number of simulations was set at 10,000, the results showed that the test had at least 0.95 power in both the direct and indirect effects for the parallel mediation model with a sample size of 600 and an alpha level of 0.05. Data were collected from a university in Wuhan City, Hubei Province, China. There were 600 participants who completed the survey regarding SNSs, social comparison, narcissism, and demographics information, i.e., age and gender, using the Wenjuanxing platform, which is an online and free-charge survey tool. The platform provided us with information about the login time of the participants on the platform and the amount of time taken to complete the questionnaire; the reaction times of all the participants were enough (*M* = 457.62s, *SD* = 78.43, ranged from 269 to 546 s). After finishing the survey, each participant was rewarded with an amount of ¥5. We used the pwrSEM app (developed by Y. Andre Wang) to make a power analysis for the mediation analysis according to the contributions of a study conducted by Wang and Rhemtulla (2021). We set the regression coefficient of each path to 0.3, which represents the moderate effect size. When the number of simulations was set at 10,000, the results showed that the test had at least 0.95 power in both the direct and indirect effects for the parallel mediation model with a sample size of 600 and an alpha level of 0.05. We then deleted 71 questionnaires with random responses, such as questionnaires in which only one answer was selected and questionnaires in which the answers were in stepped shapes. After the data sorting was administered, the final sample size was reduced to 529. Of all the participants, 133 (74.9%) were men and the mean age was 19.33 years (*SD* = 1.1). All participants were Chinese people and spoke Chinese as their mother tongue.

To benefit from the strong medical and health services provided by the motherland, most Chinese schools have opened normally since September 2020 according to the policies of the Ministry of Education. Students can study and live normally, which largely prevented the impact of the pandemic and brought benefits to our testing. Furthermore, all the participants provided informed consent before participating in the present study. They also completed all the questions online with the guidance and help of our trained psychological graduate students. We ensured the confidentiality and anonymity of the obtained responses. Meanwhile, the University Ethics Committee of our team provided the approval for the study.

### Measures

#### Surveillance Use Scale

The current study measured active and passive SNS use with a revised version of the Surveillance Use Scale by Liu et al. (2017), which was first developed by Tandoc et al. (2015). This scale has been widely used in Chinese samples, with good reliability and validity (Lian et al., 2018). Furthermore, 8 items measured the frequency of SNS use. In the scale, the study measured the active use of SNS in items 1–4, e.g., “write a status update,” and measured the passive use of SNS in items 5–8, e.g., “view a friend's photo.” We required the participants to rate each item on a scale of 1 (never) to 5 (very often). A higher score implied that the participant had a greater frequency of SNS use. The alpha coefficient of the active SNS use subscale was 0.85 and 0.84 for the passive SNS use subscale.

#### Iowa–Netherlands Comparison Orientation Measure (INCOM)

This study measured social comparison using the INCOM revised by Bai et al. (2013), which was first developed by Gibbons and Buunk (1999). However, the scope of comparison in the questionnaire was limited to “on SNS” to improve the reliability of measurement content which is according to the method in the study of Niu et al. (2016). The scale comprised 12 items with a 5-point Likert-type response from 1 (strongly disagree) to 5 (strongly agree). Notably, determining the preferred method of comparison of the participants by calculating their scores was impossible. Thus, the sample items include “In social networking sites, I often compare with others who are better than me” and “When I mess up, I often think others who do things worse than me in social networking sites.” The alpha coefficient of the upward social comparison subscale was 0.85 and 0.84 for the downward social comparison subscale.

#### Hypersensitivity Narcissism Scale (HSNS)

The present study used the HSNS to measure vulnerable narcissism, which was revised by Wang (2008) and first developed by Hendin and Cheek (1997). The scale has been widely used, with high reliability and validity (Given-Wilson et al., 2011; Brookes, 2015). The scale comprises 10 items with a 5-point Likert-type response from 1 (strongly disagree) to 5 (strongly agree). Sample items include “I feel that I am temperamentally different from most people” and “My feelings are easily hurt by ridicule or by the slighting remarks of others.” We then calculated the score of each participant and considered the higher scores of the participants as showing higher levels of vulnerable narcissism. The Cronbach's alpha for the scale in the present study was 0.72.

### Statistical Analysis

For data analysis, we performed descriptive analyses with SPSS 24 (IBM, New York). Based on our hypothesis, Pearson's correlations were used to analyze the bivariate correlations between the variables. Subsequently, we conducted mediation analyses with the PROCESS macro for SPSS (Model 4) provided by Hayes (2017), who, according to his contributions, stated that “the indirect effect of *X* on *Y* through *M*_i_ = *a*_i_ *b*_i_, and model 4 allows up to 10 mediators operating in parallel (p.7).” In addition, previous studies have revealed that individuals of different genders may differ in their performances in social comparison (Guimond et al., 2006) and narcissism (Grijalva et al., 2015). Thus, it was treated as the control variable in this study to eliminate potential confounding effects.

## Results

### Descriptive Statistics

Table 1 exhibits that all the variables were significantly correlated. Specifically, active and passive SNS use were positively associated with vulnerable narcissism (*r* = 0.104, *p* < 0.05; *r* = 0.094, *p* < 0.05), upward social comparison (*r* = 0.156, *p* < 0.001; *r* = 0.11, *p* < 0.05), and downward social comparison (*r* = 0.089, *p* < 0.05; *r* = 0.106, *p* < 0.05). Moreover, upward and downward social comparisons were positively associated with vulnerable narcissism (*r* = 0.2, *p* < 0.001; *r* = 0.255, *p* < 0.001).

**Table 1:** Descriptive statistics and inter-correlations between main variables.

**Variables**	***M***	***SD***	**1**	**2**	**3**	**4**	**5**
1 ASNS	2.613	0.608	–				
2 PSNS	3.015	0.773	0.514^***^	–			
3 USC	3.165	0.806	0.156^***^	0.110*	–		
4 DSC	2.365	0.725	0.089^*^	0.106^*^	0.186^***^	–	
5 VN	2.521	0.663	0.104^*^	0.094^*^	0.200^***^	0.255^***^	–

*N, 529. ASNS, Active social networking sites; PSNS, Passive social networking sites; USC, Upward social comparison; DSC, Downward social comparison; VN, Vulnerable narcissism*.

* *p <0.05*;

*** *p <0.001, two-tailed p for all tests*.

### Mediation Analyses

**Figure 2:**
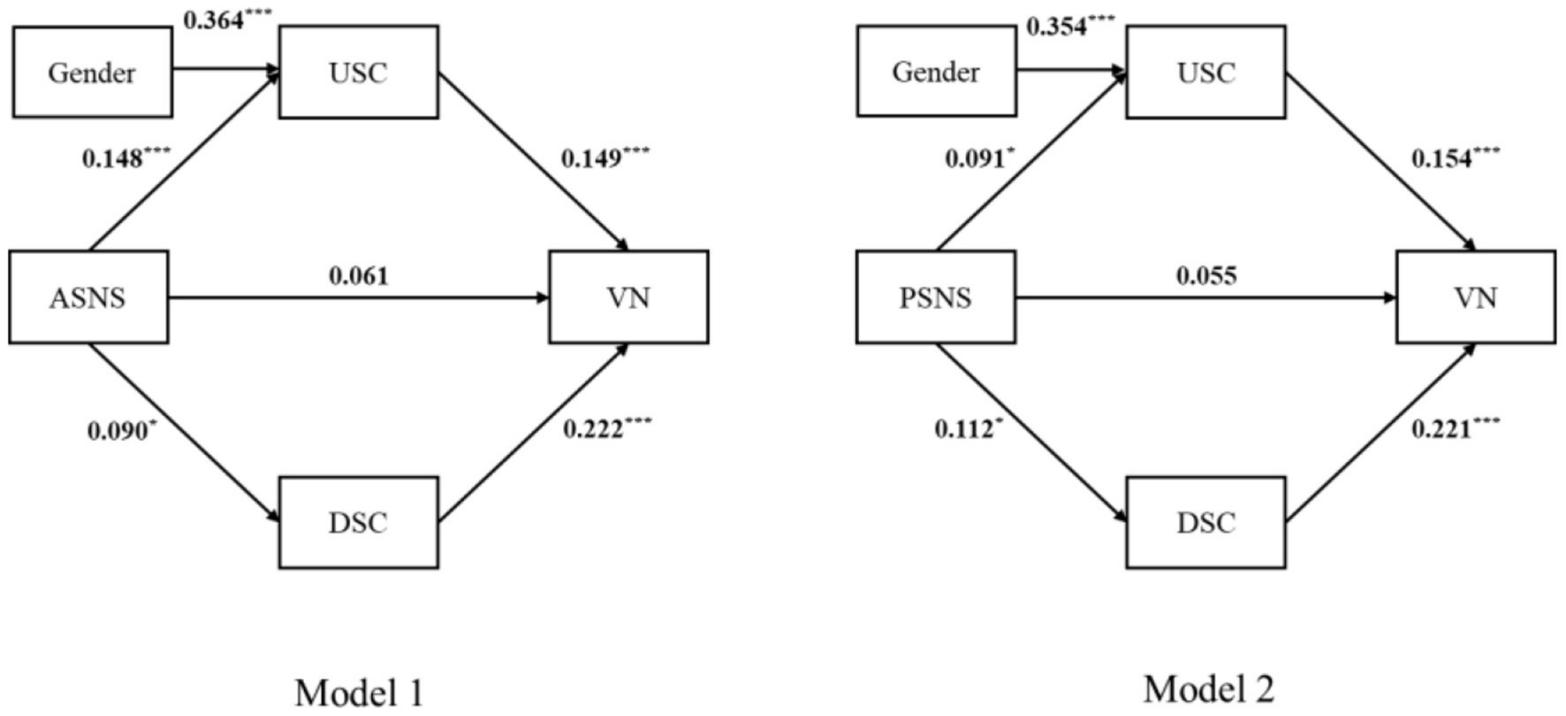
The model of the effects of active and passive social networking site use on vulnerable narcissism; Values are standardized coefficients. *p < 0.05; **p < 0.01; ***p < 0.001, two-tailed *p* for all tests; ASNS, Active social networking sites; PSNS, Passive social networking sites; USC, Upward social comparison; DSC, Downward social comparison; VN, Vulnerable narcissism. Control variable, gender.

Based on the results of the correlation analysis, we used Model 4 in the PROCESS macro to test the mediating effect. We also controlled for gender in both Model 1 and Model 2 to rule out the possible effects of those variables. The results showed that leaving out the control variable did not change the general results. Active SNS use positively predicted upward social comparison [β = 0.148, 95% CIs (0.065, 0.232), *p* < 0.001, *R*^2^ = 0.049] and downward social comparison [β = 0.09, 95% CIs (0.049, 0.176), *p* < 0.05, *R*^2^ = 0.013). Passive SNS use also positively predicted upward social comparison [β = 0.091, 95% CIs (0.006, 0.176), *p* < 0.05, *R*^2^ = 0.035] and downward social comparison [β = 0.112, 95% CIs (0.026, 0.198), *p* < 0.05, *R*^2^ = 0.013]. In Model 1, mediation analyses found that upward and downward social comparisons served as the predictors for vulnerable narcissism [β = 0.149, 95% CIs (0.064, 0.234), *p* < 0.001; β = 0.222, 95% CIs (0.139, 0.306), *p* < 0.001; *R*^2^ = 0.093], but active SNS did not predict vulnerable narcissism (β = 0.061, *p* > 0.05) (as shown in Figure 2). The results also revealed that the indirect relationships between active SNS use and vulnerable narcissism through upward and downward social comparisons were significant [95% CIs (0.006, 0.044), (0.001, 0.043)] (as shown in Table 2). In Model 2 (as shown in Figure 2), upward and downward social comparisons served as the predictors for vulnerable narcissism [β = 0.154, 95% CIs (0.069, 0.238), *p* < 0.001; β = 0.221, 95% CIs (0.137, 0.304), *p* < 0.001; *R*^2^ = 0.092], but passive SNS did not predict vulnerable narcissism (β = 0.055, *p* > 0.05). The indirect relationships between passive SNS use and vulnerable narcissism through upward and downward social comparisons were also significant [95% CIs (0.001, 0.032), (0.006, 0.047)] (as shown in Table 2). Therefore, upward and downward social comparisons act as full mediators in Model 1 and Model 2.

**Table 2:** Indirect effects with upward and downward social comparisons as mediators.

		**Indirect effect**	**Boot SE**	**Boot 95% CI**	
				**LL**	**UL**
Model 1	Total indirect effect	0.042	0.015	0.015	0.072
	USC	0.022	0.010	0.006	0.044
	DSC	0.020	0.011	0.001	0.043
	USC-DSC	0.002	0.015	−0.026	0.031
Model 2	Total indirect effect	0.039	0.013	0.016	0.065
	USC	0.014	0.008	0.001	0.032
	DSC	0.025	0.011	0.006	0.047
	USC-DSC	−0.011	0.013	−0.038	0.015

*N, 529. Bootstrap resample size, 5,000; SE, Standard error; LL, Lower limit; UL, Upper limit; CI, Confidence interval. Control variable, gender*.

## Discussion

### Social Networking Site Use and Vulnerable Narcissism

This study showed that active or passive SNS use did not significantly relate to vulnerable narcissism. However, active and passive SNS use can indirectly predict the vulnerable narcissism of an individual. The findings of the present study were inconsistent with previous research results, which found that active and passive SNS use have different effects on individuals (Chen et al., 2016; Wang et al., 2018). These results are not surprising. As some researchers have pointed out, most media effects are indirect rather than direct, which means that we need to specify the boundary conditions of media effects (Valkenburg et al., 2016). Intervening variables should not be ignored even if we cannot assert the impact of SNS use on narcissism just because SNS provide opportunities for high-level narcissists to improve themselves and seek attention (McKinney et al., 2012; Walters and Horton, 2015). The mediating mechanism can provide essential explanations on how and why media effects occur. Therefore, this mediating mechanism can be helpful in establishing prevention and intervention programs.

### Mediating Role of Social Comparison

The result revealed that upward and downward social comparisons are the parallel mediating variables in the relationship between SNS use and vulnerable narcissism, which supports Hypothesis 2. Both active and passive SNS use could show pretty much identical relationships in both models. On the one hand, both active and passive SNS use could significantly predict social comparison. First, individuals publish a lot of information about themselves on SNSs; simultaneously, they inevitably become the audience for other users when posting content (Vogel et al., 2014). Therefore, the social comparison seems inevitable in the process of using SNSs. In addition, individuals actively choose one or a certain class of objects for comparison according to different purposes, e.g., individuals choose upward social comparison to motivate themselves or deliberately choose downward social comparison to maintain a good sense of themselves. As pointed out by the study of Festinger (1954), social comparison is a process by which individuals actively seek relevant information from others to obtain an accurate self-evaluation. The positive information of others and the visibility of the feedback presented on SNS encourage individuals to make upward social comparisons (Fox and Vendemia, 2016). Moreover, in the absence of motivation for an individual to perform active comparisons, a social comparison may happen automatically as long as the information of others is presented (Mussweiler and Rüter, 2003; Chatard et al., 2017). Previous studies have indicated that passive SNS usage is positively associated with upward social comparison (Lee, 2014; Zheng et al., 2020), and the results revealed that social comparison may be an unconscious and spontaneous behavior. Thus, a social comparison might happen automatically in the process of obtaining the information of other people regardless of the individuals use the network actively or passively; this is especially evident to those who spend more time on social networks (Lee, 2014). Hence, active SNS use and passive SNS use showed similar patterns among different models.

On the other hand, the results showed that both upward social comparison and downward social comparison could significantly predict vulnerable narcissism. There are at least two reasons that can explain this phenomenon, one being the assimilation effect on positive information. Many previous studies have focused on the negative effects of upward social comparison on individuals, e.g., Pang (2021). However, few studies have found that upward social comparison can be used as a strategy for the long-term self-improvement of individuals, i.e., Michinov and Bavent (2001). Thus, when an individual faces social comparison information, the self-evaluation level displaces toward the comparison goal (Collins, 1996). For example, a study on Twitter found that users might post more tweets after viewing the positive tweets of others (Ferrara and Yang, 2015). Furthermore, it has been found that upward social comparisons promote the highest levels of motivation for self-improvement compared with downward social comparisons (Peng et al., 2019). Therefore, an individual may show their narcissism when they increase their self-evaluation level when facing upward comparison information. The second reason for both upward and downward social comparisons significantly predicting vulnerable narcissism is the contrast effect induced by the negative information of other people. According to the previous findings, compared with people who had fewer likes on social networks, participants experienced more positive emotions (Rosenthal-von der Pütten et al., 2019) and vulnerable narcissists had the tendency to pay great attention to themselves, eager to be affirmed and praised by others (Pincus and Lukowitsky, 2010). Moreover, the present study also found a significant positive correlation between downward social comparison and vulnerable narcissism, which was consistent with the previous studies (Kong et al., 2020). Thus, the social comparison makes efforts in narcissism.

### Limitations and Directions

This study holds certain limitations. First, active and passive SNS use exist in social networking activities simultaneously, which may entail a mutual influence. The personalities and beliefs of individuals may influence their choices of information and communication style. In turn, media use may also affect individual attitudes and behaviors. Future research can use a longitudinal design to investigate the relationship between SNSs and vulnerable narcissism in a dynamic interaction process. Second, the current study used a self-report method, in which some social desire effects may exist. Future studies can explore the relationship between SNSs and vulnerable narcissism in behavioral and neuroscience experiments. Third, due to the inherent imperfections of statistical procedures, the alpha correction, and other statistical methods, e.g., Bayesian analysis, should be considered to clarify the studied relationship further.

## Conclusions

This study demonstrated that close relationships exist between SNS use and social comparison and vulnerable narcissism. Moreover, upward and downward social comparisons were the parallel mediators in the relationship between active or passive SNS use and vulnerable narcissism.

## Data Availability Statement

The raw data supporting the conclusions of this article will be made available by the authors, without undue reservation.

## Ethics Statement

The studies involving human participants were reviewed and approved by Ethics Committee of Central China Normal University. Written informed consent to participate in this study was provided by the participants' legal guardian/next of kin.

## Author Contributions

FK conceived the idea and conducted the literature searches. MW and FK wrote and revised the manuscript. XZ and XL collected the research data and performed the statistical analysis. XS polished the language of this manuscript. All the authors read and approved the submitted version.

## Funding

This work was supported by the Ministry of Education of Humanities and Social Science project (17YJC190008), the Research Program Funds of the Collaborative Innovation Center of Assessment Toward Basic Education Quality at Beijing Normal University (KJ02252019-0801), Fundamental Research Funds of Central China Normal University (CCNU19QN042), and fund for building world-class universities (disciplines) of Renmin University of China (RUCPSY0001). The funders had no role in study design, data collection and analysis, decision to publish, or preparation of the manuscript.

## Conflict of Interest

The authors declare that the research was conducted in the absence of any commercial or financial relationships that could be construed as a potential conflict of interest.

## Publisher's Note

All claims expressed in this article are solely those of the authors and do not necessarily represent those of their affiliated organizations, or those of the publisher, the editors and the reviewers. Any product that may be evaluated in this article, or claim that may be made by its manufacturer, is not guaranteed or endorsed by the publisher.
